# Editorial: Errors and biases in modern healthcare: public health, medico-legal and risk management aspects

**DOI:** 10.3389/fmed.2025.1676522

**Published:** 2025-08-29

**Authors:** Simone Grassi, Francesco Ausania, Davide Ferorelli, Francesco De Micco

**Affiliations:** ^1^Department of Health Science, Section of Forensic Medical Sciences, University of Florence, Florence, Italy; ^2^Department of Diagnostics and Public Health, Section of Forensic Medicine, University of Verona, Verona, Italy; ^3^Section of Legal Medicine, Department of Interdisciplinary Medicine, University of Bari, Bari, Italy; ^4^Research Unit of Bioethics and Humanities, Department of Medicine and Surgery, Università Campus Bio-Medico di Roma, Rome, Italy

**Keywords:** risk management, legal medicine, medical error, medical bias, medical malpractice

Is to err (only) human nowadays? Error is traditionally considered an unavoidable part of human life and activities, and the phrase “to err is human” has been a sort of dogma in medicine for the last decades (1). The actual genesis of human error is still largely unexplored, and the interplay between hospital organization, enforcement of policies, and human choices is still far from being decrypted (2, 3). However, we do know that teamwork is the bedrock of modern medicine and relies on human paradigms like trust (4). Can we trust artificial intelligence (AI)? The question is misleading. AI is a technology able to find correlations between input variables, but it does not currently have the capacity to infer causal relationships (at least following the traditional rules of logic). AI has no professionality, no free will, no reputation: physicians must learn how to work with a technology suppressing the human need to trust it and how to supervise its outputs often without the possibility to understand its internal processes (4). A new kind of error—the bias (systematic error)—will emerge as a leading issue in medicine. Improving in-hospital management processes is thus the most pragmatic choice that the institutions can make now. But AI is not only a source of new kinds of errors: it does help to contain common risks in diagnosis, treatment, and prevention programs (5–7). Therefore, a constructive and pragmatic attitude is needed.

One of the biggest challenges in modern healthcare is linked to healthcare-acquired infections (HAI): multidrug-resistant bacteria are a public health threat, and HAI inflate in-hospital mortality, length of stays, direct costs of care, and disability-adjusted life years (8).

Ferorelli et al. evaluated how a new clinical risk management protocol impacted newborns, finding a reduction in umbilical venous catheter infections and an improvement in hospital stay lengths. On the other hand, exploring medical malpractice claims for HAI, Grassi, Grazzini et al. performed a cost-effectiveness analysis using epidemiological data, including experts in infection prevention and control sitting on the hospital decisional committee. They found that improving the management of medical malpractice claims can improve the economic outcomes, containing the double cost (implementing policies against HAI and paying for the related claims) that many hospitals must face because of a public health issue (Grassi, Grazzini et al.). De Micco, Grassi et al. stressed that new technologies have already been introduced in many hospitals, but regulations are still vague and insufficient, with the borders between the liability of the manufacturer/developer, the institution, and the user still ill-defined. Instead, Kameyama et al. evaluated the impact on the drug market of a Japanese risk management plan, finding that, when specific adverse reactions and drug therapeutic categories were evaluated, it succeeded in improving patient safety.

Moreover, our special issue hosted a systematic review on the potential role of AI systems to promptly detect adverse events (making correlations that a human mind could not make or would take a longer time to make), predict future incidents, and assess risks of sentinel events like in-hospital falls (De Micco, Di Palma et al.). Regarding sentinel events, another contribution evaluated the actual impact of retained surgical foreign bodies impact, finding that, despite their health consequences, they are generally mild and have a 2-fold risk of criminal complaint, indicating that the patient blames specific professionals rather than the (impersonal) institution for the events (Grassi, Focardi et al.). Visci et al. focused on a topic of utmost relevance: can the knowledge on the causal processes related to medical errors developed by the experts in legal medicine be used to improve clinical services? Their observational study concluded that structured forensic consultation services should be incorporated into clinical practice, being able to quickly and professionally address hot topics like capacity for consent that are frequent issues that can “paralyze” hospital activities (Visci et al.). Refolo et al. analyzed another face of the term “risk”: the risk of aggressive behavior based on genetic predisposition. Aggressivity is an issue of public interest both in hospital and common social contexts, and the potential role of genes in risk profiling has a potential dual use that should lead to strong ethical barriers (Refolo et al.). Finally, Aurilio et al. discussed the birth-related long bone fractures in healthy newborns—an example of an adverse event that can be both due to unavoidable factors and to human errors, with the compliance with best practices and prevention policies being the cornerstone of medico-legal defense.

In conclusion, in medicine, there is no such thing as a rigid dichotomic difference between errors and proper conduct, but there is a risk density entangled with medical services that can be addressed/neglected, inflated/deflated, or defined/left unknown. Hunting for manipulable variables in the unknown and improving the management of the risks are the real keywords of the papers of this special issue. To err is not only human anymore, but only humans can envision the future in healthcare and tailor new strategies to make it safe for all stakeholders.
